# Hospital variations in caesarean delivery rates: An analysis of national data in China, 2016-2020

**DOI:** 10.7189/jogh.13.04029

**Published:** 2023-04-07

**Authors:** Shaohua Yin, Yubo Zhou, Pengbo Yuan, Yuan Wei, Lian Chen, Xiaoyue Guo, Hongtian Li, Jie Lu, Lin Ge, Huifeng Shi, Xiaoxia Wang, Luyao Li, Jie Qiao, Dunjin Chen, Jianmeng Liu, Yangyu Zhao

**Affiliations:** 1 Department of Obstetrics and Gynecology, Peking University Third Hospital, Beijing, China; 2 National Centre for Healthcare Quality Management in Obstetrics, Peking University Third Hospital, Beijing, China; 3 National Clinical Research Centre for Obstetrics and Gynecology, Peking University Third Hospital, Beijing, China; 4 Institute of Reproductive and Child Health, Peking University Health Science Centre, Beijing, China; 5 National Health Commission Key Laboratory of Reproductive Health, Peking University Health Science Centre, Beijing, China; 6 Department of Obstetrics and Gynecology, the Third Affiliated Hospital of Guangzhou Medical University, Guangzhou, China

## Abstract

**Background:**

The impact of China’s use of caesarean delivery on global public health has been a long-term concern. The number of private hospitals is increasing in China and likely driving up caesarean delivery rates, yet specifics remain unknown. We aimed to investigate variations in caesarean delivery rates across and within hospital types in China.

**Methods:**

We retrieved data on hospital characteristics and national hospital-level annually aggregated data on the number of deliveries and caesarean deliveries from 2016-2020, covering 7085 hospitals in 31 provinces of mainland China, from the National Clinical Improvement System. We categorized hospitals as public-non-referral (n = 4103), public-referral (n = 1805) and private (n = 1177). Among the private hospitals, 89.1% (n = 1049) were non-referral regarding obstetrical services for uncomplicated pregnancies.

**Results:**

Among 38 517 196 deliveries, 16 744 405 were caesarean, giving an overall rate of 43.5% with a minor range of 42.9%-43.9% over time. Median rates differed across hospital types, from 47.0% (interquartile range (IQR) = 39.8%-55.9%) in public-referral, 45.8% (36.2%-55.8%) in private, and 40.3% (30.6%-50.6%) in public-non-referral hospitals. The stratified analyses corroborated the results, except for the northeastern region, where the median rates did not differ across the public-non-referral (58.9%), public-referral (59.3%), and private (58.8%) hospitals, while all ranked higher than the other regions, regardless of hospital type and urbanization levels. The rates within hospital types differed as well, especially in the rural areas of the western region of China, where the difference of rates between the 5th and 95th percentiles was 55.6% (IQR = 4.9%-60.5%) in public-non-referral, 51.5% (IQR = 19.6%-71.1%) in public-referral, and 64.6% (IQR = 14.8%-79.4%) in private hospitals.

**Conclusions:**

Variation across hospital types in China was pronounced, with the highest rates either in public-referral or private hospitals, except in the northeastern region, where no variation was observed among the high rates of caesarean deliveries. Variation within each hospital type was pronounced, especially in rural areas of the western region.

Both caesarean overuse and underuse can predispose mothers and children to health risks [1], even complicating future pregnancies [2]. Caesarean delivery rates in China have historically been close to those in the western world [3]. Given that a large portion of the global population lives in China, the impact of caesarean delivery on global public health has been a constant concern [4-6].

In 2022, China was still the most populous country globally, and was second in birth rates, overtaken by India [4]. Trends in China can alter the dynamics of global health metrics [5], especially those related to its health policies or health care system. Now, almost all deliveries in mainland China occur in hospitals [7], indicating that they might play a role in the overuse of caesarean delivery. This role might differ with hospital types, such as public-referral, public-non-referral, and private hospitals. For example, public-referral hospitals have a higher capacity for dealing with complicated pregnancies that are more likely to end with a caesarean delivery, as compared with public non-referral hospitals and private hospitals that are overwhelmingly non-referral [8]. China permitted private investments into the health care industry in 2000 [9] and further encouraged them in 2009 [10] to expand health care supply and to narrow the regional gap in health care access, leading to a rise in the number of private hospitals [8]. By 2020, a variety of private hospitals (including maternity hospitals), neither operated nor funded by governmental authorities, accounted for 66% of the number of total hospitals, but received merely 16% of the total patient visits [11]. This trend is similar to the one in India [12] but differs from western nations. In India, private hospitals account for 63% of all hospitals and serve 26% of patients. In the USA, private hospitals encompass 68% of all hospitals and serve 80% of the patients [13], while these figures are much lower in the UK [14], at 20% and 10%, respectively. China’s private maternity hospitals, usually having a better environment, better hospitality, decreased waiting time, meticulous management, and bigger expenses compared with public hospitals, have been emerging and expanding in the last two decades; most of them are non-referral, providing services for patients with uncomplicated pregnancies who pay out of pocket and are not reimbursed by governmental health insurances [8]. Thus, for-profit private hospitals are more likely to provide caesarean procedures than public hospitals. Significant differences in caesarean delivery rates between private and public hospitals have been previously reported in India, Brazil, and Australia [12,15,16], but no such data are available for China [3,5,17]. Furthermore, the universal two- and three-child policies commenced in 2015 and 2021 allowed couples to have two to three children and have been speculated to have helped promote vaginal delivery and discourage the overuse of caesarean deliveries in hospitals with high rates [18], but there is a lack of data on this phenomenon in public-nonreferral, public-referral and private hospitals [3,19]. Such data could provide a basis for domestic policy-making amid high in-hospital delivery, but could have importance for the global challenge regarding the over- or under-use if caesareans deliveries.

The National Clinical Improvement System, established by the National Health Commission of China, started collecting national hospital-level data on hospital attributes, caesarean delivery, maternal death, and other obstetric-related indicators in 2016. We aimed to investigate variations in caesarean rates across- and within-hospital types, hypothesizing that the rates were higher in public-referral and private hospitals than the public-nonreferral, with marked variations in each type of hospitals. Additionally, we attempted to investigate the characteristics of hospitals where high or low rates were present.

## METHODS

### Data sources

We used hospital-level annual aggregated data from the National Clinical Improvement System (NCIS), a national data collection platform established by the National Health Commission of China in 2011, aiming to improve the quality of clinical services in China. The NCIS platform was designed with strict quality control procedures to ensure the integrity, logicality, and accuracy of data entry. Any potential erroneous data would be returned to hospitals for further checking and necessary corrections. The hospitals were selected annually through a consistent, multistage, stratified sampling strategy, to be representative at national, provincial, prefecture, and county levels (Table S1 in the **Online Supplementary Document**). The aggregated data reported by trained staff working at the selected hospitals were derived from the front page of inpatient records. In 2016, the NCIS started to collect obstetric service metrics, including a range of maternal and perinatal health indicators.

### Data extraction and cleaning

We extracted the number of live births by caesarean delivery, total live births and total deliveries for 2016 and for the 2017-2020 period from the NCIS data set. We also extracted other annual data on: maternal death, women with epidural anaesthesia, women with severe morbidity, women with advanced maternal age (AMA) (≥35 years), multiparous women, women with multiple pregnancies, macrosomia, and midwives. We also extracted information on hospital attributes, including hospital ownership (public- or private-owned) and hospital level (referral or nonreferral).

We then categorized the hospitals as public-nonreferral, public-referral, and private. Only 10.9% (128/1177) of the private hospitals were referral. In the preliminary analysis, caesarean delivery rate in private-referral hospitals (44.1%) appeared similar to that in private-nonreferral hospitals (45.9%), so we combined them in the formal analyses. We also extracted the hospitals’ geographic information, including province, city district, or county. According to the National Bureau of Statistics of China [20,21], there are four geographic regions (northeastern, eastern, central, and western regions of mainland China) and two areas (rural (counties) and urban areas (cities)); in case of the latter, a “supercity” refers to a city with a population of five million, while the others are categorized as general cities [22].

We calculated hospital-specific percentages for multiparous women as the number of multiparous women divided by the number of total deliveries occurring at a hospital, and performed the same calculation for other maternal characteristics. We calculated hospital-specific percentages of macrosomia cases as the number of macrosomia cases divided by the number of total live births occurring at a hospital, and the hospital-specific density of midwives as ratio of the number of midwives and the total live births at a hospital.

### Caesarean delivery rates

We calculated caesarean delivery rates as the number of caesarean deliveries divided by the number of total deliveries. In 2016, the number of live births by caesarean delivery was collected but not total number of caesarean deliveries. The temporal trends in caesarean deliveries and corresponding rates during 2017-2020 were linear or stable in most hospitals, so we established liner regression models by using 2017-2020 data for each hospital to estimate the missing data for 2016. The estimated 2016 caesarean delivery rate (42.9%) extrapolated from the total number of caesarean deliveries did not materially differ from the estimated rates based on live births by caesarean deliveries (43.0%), and neither did those in the following years (Table S2 in the **Online Supplementary Document****)**.

### Statistical analyses

We reported categorical variables as number and percentages and examined the differences in maternal, hospital, and geographic characteristics across the three hospital types using χ^2^ tests. We reported continuous variables as means (standard deviation (SDs)) or medians (interquartile ranges (IQRs)) depending on their distributions assessed by the Shapiro-Wilk normality test, and examined the differences by analysis of variance or Kruskal-Wallis tests, as appropriate. We calculated hospital-specific caesarean delivery rates annually to examine the trend over time. Given the steady rate, we calculated the hospital-specific rate by using the total number of deliveries occurring at a specific hospital over the whole study period to increase robustness for primary analysis on hospital variations. To characterize the across- or within-hospital-type variations, we calculated medians with IQRs or differences between the 5th and 95th percentiles of caesarean delivery rates. We generated box-and-whisker plots to visualize the variation over subgroups of hospitals defined by geographic region (northeastern, eastern, central, and western regions), urbanization level (supercity, general city and rural areas), hospital-specific percentage of AMA women (terciles), hospital-specific percentage of multiparous women (terciles), hospital-specific percentage of women with multiple pregnancies (terciles), hospital-specific percentage of macrosomia cases (terciles), hospital-specific percentage of women with epidural anaesthesia, and 8) hospital-specific annual delivery volume (100-999, 1000-2999, 3000-4999, and ≥5000 deliveries).

We established linear regression models with bootstrapping to explore potential sources of the hospital variations, including maternal factors (hospital-specific percentage of AMA women, multiparous women, women with multiple pregnancies, and macrosomia, maternal mortality, and maternal severe morbidity rate), hospital factors (hospital-specific percentage of women with epidural anaesthesia, density of midwives, and hospital-specific annual delivery volume), and geographic factors (provinces, cities within province, and urbanization level). We incorporated individual factors which were statistically significant (*P* < 0.05) in univariate models into multivariate models according to hospital types, to calculate the coefficient of partial determination (partial *R*^2^) for each of incorporated factors. The partial *R*^2^ indicated the percentage of variances in hospital-level rates accounted for by a given factor.

We performed all analyses using SAS version 9.4 (SAS Institute Inc), setting the statistical significance at a two-sided *P* < 0.05.

### Patient and public involvement

Patients and the public were not involved in the design or conduct of our research.

## RESULTS

### Cross-validation of the NCIS caesarean delivery rates

We validated the NCIS caesarean delivery rates against the rates from 438 hospitals in 30 provinces of mainland China reported to the National Maternal Near Miss Surveillance System (NMNMSS) [23,24], an independent system collecting obstetric data. The 2016 rate was 42.9% in our NCIS data and 42.1% in the NMNMSS data [23], while the 2018 rates 43.4% and 45.0%, respectively [24]. The included hospitals and targeted goals differed substantially between the two systems, but the caesarean rates did not.

### Main findings

Among 20 408 hospital-year records initially extracted from the NCIS for the 2016-2020 period, we excluded records with missing data on live births by caesarean delivery, those with obvious data entry errors, or records for hospitals in which there were less than 100 annual deliveries. Finally, we included 18 197 hospital-year records involving 38 517 196 deliveries from 7085 hospitals spreading throughout 31 provinces of mainland China (Figure S1 in the **Online Supplementary Document**).

**Figure 1:**
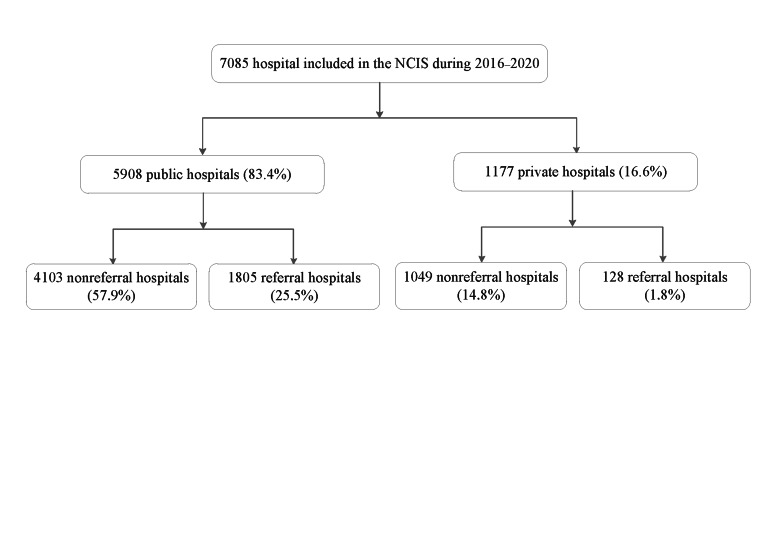
Distribution of hospital types in the NCIS, 2016-2020.

**Figure 2:**
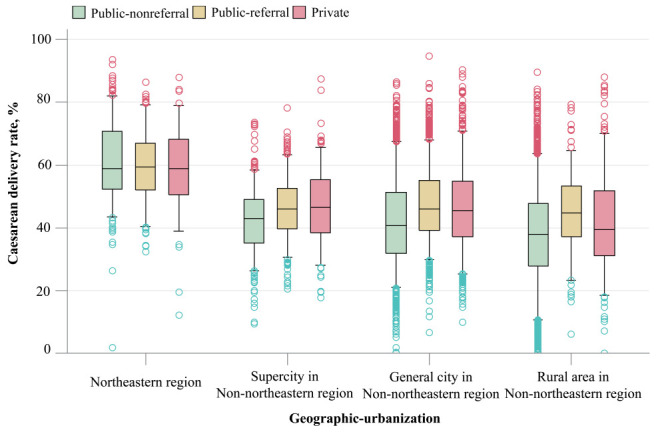
Caesarean delivery rates across hospital type by geographic region and urbanization level, 2016-2020. For each box-and-whisker plot, the horizontal bar indicates the median, the upper and lower limits of the boxes the interquartile range, and the ends of the whiskers from bottom of box to top indicate the 5th percentile and the 95th percentile. The green and red circles respectively represent the rates less than the 5th percentile or greater than the 95th percentile.

Of the 7085 hospitals, 4103 (57.9%) were public-non-referral, 1805 (25.5%) public-referral, and 1177 (16.6%) private; among the private ones, 1049 (89.1%) were non-referral (**Figure 1**). Of the 38 517 196 deliveries, 16 482 849 (42.8%) occurred in public-non-referral hospitals, 19 062 308 (49.5%) in public-referral hospitals, and 2 971 967 (7.7%) in private hospitals (**Table 1**). While they had the highest macrosomia rates, the highest number of midwives, the highest proportion of vaginal deliveries with epidural anaesthesia, and the lowest maternal mortality rates, private hospitals were similar to public-non-referral hospitals in other characteristics, including advanced maternal age, nulliparity, multiple pregnancies and severe morbidities. These characteristics, together with maternal deaths, were most likely to be observed in public-referral hospitals (**Table 1**). Among the total deliveries, 16 744 405 were caesareans, giving an overall caesarean delivery rate of 43.5% with a minor range of 42.9%-43.9% from 2016 to 2020 (Table S2 in the **Online Supplementary Document**). The median caesarean rates varied across hospital types and was the highest in public-referral (47.0%, IQR = 39.8%-55.9%), followed by private hospitals (45.8%, IQR = 36.2%-55.8%) and public-non-referral hospitals (40.3%, IQR = 30.6%-50.6%) (**Table 2**). Analysis stratified by maternal, hospital, and geographic characteristics consistently showed the lowest rate in public-non-referral hospitals and the highest in public-referral or private hospitals (Figure S2 in the **Online Supplementary Document**). Notably, when stratified by geographic region, an exception occurred in the northeastern region of China, where median caesarean rates did not materially vary across public-non-referral hospitals (58.9%, IQR = 52.3%-70.8%), public-referral hospitals (59.3%, IQR = 52.0%-67.0%), and private hospitals (58.8%, IQR = 50.4%-68.1%); these three respective rates were the highest compared to other regions (Figure 2A the **Online Supplementary Document**), and were all markedly higher than those even for 14 super cities in other regions of China, not to mention for general cities or rural counties (**Figure 2**). Despite the extremely high caesarean rates in the northeastern region of China, its maternal mortality rates remained higher compared to other regions (Figure S3 in the **Online Supplementary Document**). Maternal mortality rates for public-referral hospitals were 11.2 per 100 000 live births in the northeastern region and 7.1 per 100 000 live births in the other regions, and corresponding rates were 4.6 and 4.0 per 100 000 live births, respectively, for public-non-referral and private hospitals.

**Table 1:** Maternal, hospital, and geographic characteristics by hospital types, 2016-2020*

		Hospital types		
	**Total**	**Public-non-referral**	**Public-referral**	**Private**
**Characteristic**	**(n = 38 517 196)**	**(n = 16 482 849)**	**(n = 19 062 380)**	**(n = 2 971 967)**
**Maternal age**				
<35	3 3759 189 (87.8)	14 739 728 (89.4)	16 360 348 (85.8)	2 659 113 (89.5)
≥35	4 758 007 (12.2)	1 743 121 (10.6)	2 702 032 (14.2)	312 854 (10.5)
**Parity**				
Nulliparity	18 010 527 (46.8)	6 973 612 (42.3)	9 702 507 (50.9)	1 334 408 (44.9)
Multiparity	20 506 669 (53.2)	9 509 237 (57.7)	9 359 873 (49.1)	1 637 559 (55.1)
**Plurality of birth**				
Singleton	37 953 980 (98.5)	16 343 290 (99.2)	18 665 921 (97.9)	2 944 769 (99.1)
Multiple	563 216 (1.5)	139 559 (0.8)	396 459 (2.1)	27 198 (0.9)
**Maternal deaths†**	1802 (5.7)	586 (4.3)	1151 (7.3)	65 (2.7)
**Maternal severe morbidity‡**	173 531 (0.7)	36 890 (0.3)	130 189 (1.1)	6452 (0.3)
**Macrosomia (birthweight ≥4000 g)§**	2 251 406 (5.8)	963 153 (5.8)	1 080 474 (5.6)	207 779 (7.0)
**Epidural anaesthesia‖**	4 379 605 (24.5)	1 511 704 (18.5)	2 420 835 (28.9)	447 066 (34.1)
**Density of midwives¶**	6.7 (213 045/31 853 275)	7.2 (98 798/13 715 596)	6.0 (93 653/15 739 589)	8.6 (20 603/2 398 090)
**Hospital delivery volume as deliveries/y, median (IQR)**	1188 (506-2387)	1088 (497-2049)	2294 (1093-3994)	569 (268-1251)
**Geographic region**				
Northeastern	1 660 255 (4.3)	442 939 (2.7)	1 056 200 (0.9)	161 116 (5.4)
Eastern	17 853 494 (46.4)	6 830 411 (41.4)	9 574 601 (8.0)	1 448 482 (48.7)
Central	8 683 875 (22.5)	4 316 057 (26.2)	3 507 539 (2.9)	860 279 (28.9)
Western	10 319 572 (26.8)	4 893 442 (29.7)	4 924 040 (4.1)	502 090 (16.9)
**Urbanization level**				
Supercity	6 976 455 (18.1)	1 676 172 (10.2)	4 693 368 (24.6)	606 915 (20.4)
General city	21 052 433 (54.7)	6 255 242 (38.0)	13 082 530 (68.6)	1 714 661 (57.7)
Rural	10 488 308 (27.2)	8 551 435 (51.9)	1 286 482 (6.7)	650 391 (21.9)

AMA – advanced maternal age, y – year, g – grams, IQR – interquartile range

*Unless otherwise indicated, data are expressed as number (percentage) of deliveries. Percentages may not sum to 100 due to rounding.

†Data in parentheses are expressed as average number of maternal deaths per 100 000 live births; the number of maternal deaths is not available for 2016.

‡Maternal severe morbidity included eclampsia, acute renal failure, acute respiratory failure, cardiac arrest, pulmonary oedema, cardio-cerebrovascular diseases, epilepsy, anaphylactic shock, anaesthesia accident, etc.; the number of women with severe morbidity is not available for 2016 and 2020.

§Data are expressed as number of macrosomia, and data in parentheses are expressed as average number of macrosomia per 100 live births.

‖Data are expressed as number of women with epidural anaesthesia, and data in parentheses are expressed as average number of women with epidural anaesthesia per 100 vaginal deliveries; the number of women with epidural anaesthesia is not available for 2016.

¶Data are expressed as average number of midwives per 1000 live births; the number of midwives is not available for 2016.

**Table 2:** Percentiles of caesarean delivery rates by hospital types, 2016-2020

	Percentiles					
	**5th**	**25th**	**50th**	**75th**	**95th**	Difference*
**Total hospital caesarean rate (%)**	19.7	33.8	43.3	53.4	68.8	49.1
Public-nonreferral hospitals	15.9	30.6	40.3	50.6	67.9	52.0
Public-referral hospitals	29.7	39.8	47.0	55.9	69.2	39.5
Private hospitals	24.4	36.2	45.8	55.8	71.5	47.1

*Calculated by subtracting the 5th percentile from the 95th percentile.

When stratified by province, we observed the highest rate in Heilongjiang province, one of three northeastern provinces, with a median caesarean delivery rate of 64.3% (IQR = 56.9%-73.7%) in public-non-referral hospitals, 62.5% (IQR = 56.3%-68.8%) in public-referral hospitals, and 63.2% (IQR = 56.1%-72.8%) in private hospitals. When stratified by a combination of geographic region and urbanization level, we observed lowest rate in public-non-referral hospitals in rural areas of the western region of China (**Table 3**), particularly in Tibet, Yunan, Gansu, Qinghai, Guangxi and Ningxia provinces, where the median caesarean delivery rate was 22.0% (IQR = 11.5%-30.0%) in public-non-referral hospitals, 22.5% (IQR = 18.3%-27.1%) in public-referral hospitals, and 27.8% (IQR = 22.1%-39.6%) in private hospitals.

**Table 3:** Median and difference of caesarean delivery rates across hospital types by geographic region and urbanization level, 2016-2020

		Public-nonreferral hospitals		Public-referral hospitals		Private hospitals	
**Geographic region**	**Urbanization level**	**Median (IQR)**	Difference (5th-95th percentile)*	**Median (IQR)**	Difference (5th-95th percentile)*	**Median (IQR)**	Difference (5th-95th percentile)*
Northeastern†	General city	59.3 (53.1-72.3)	40.2 (43.9-84.1)	59.4 (52.0-67.8)	37.1 (42.3-79.4)	58.1 (49.9-64.8)	37.9 (38.9-76.8)
	Rural	58.4 (50.6-69.3)	38.2 (41.5-79.7)	55.1 (47.5-60.3)	24.6 (40.3-64.9)	65.4 (57.1-72.8)	31.6 (56.2-87.8)
Eastern	Supercity	44.3 (38.2-50.4)	28.4 (31.3-59.7)	46.7 (42.3-53.0)	29.5 (33.2-62.7)	41.6 (35.5-54.6)	39.9 (27.3-67.2)
	General city	38.2 (31.2-49.2)	44.9 (21.8-66.7)	42.9 (37.4-50.6)	33.4 (30.3-63.7)	45.2 (37.4-54.6)	48.6 (24.5-73.1)
	Rural	41.6 (32.6-50.5)	45.4 (21.6-67.0)	41.3 (37.0-45.9)	28.1 (26.9-55.0)	44.6 (35.4-53.8)	44.9 (25.1-70.0)
Central	General city	45.0 (36.6-53.4)	43.0 (26.2-69.2)	49.8 (41.8-58.5)	34.9 (34.8-69.7)	46.2 (39.5-55.0)	39.1 (28.1-67.2)
	Rural	40.3 (32.1-49.5)	42.4 (20.6-63.0)	42.9 (36.3-50.1)	29.9 (29.1-59.0)	37.9 (30.5-50.1)	46.0 (19.5-65.5)
Western	Supercity	46.7 (42.7-50.6)	18.9 (35.7-54.6)	47.8 (45.0-52.6)	21.5 (41.5-63.0)	54.9 (49.0-59.8)	24.0 (43.5-67.5)
	General city	38.1 (28.8-49.8)	48.2 (16.0-64.2)	46.8 (38.9-55.7)	42.4 (26.0-68.4)	44.0 (33.7-54.4)	47.5 (23.5-71.0)
	Rural	32.5 (22.2-43.9)	55.6 (4.9-60.5)	50.8 (42.5-57.7)	51.5 (19.6-71.1)	38.4 (28.4-50.9)	64.6 (14.8-79.4)

IQR – interquartile range

*Calculated by subtracting the 5th percentile from the 95th percentile.

†No available data from supercity located in northeastern region.

There was also a large variation in caesarean delivery rates within hospital types. The difference between the 5th to 95th percentiles of caesarean delivery rates was 52.0% (IQR = 15.9%-67.9%) in public-non-referral hospitals, 39.5% (IQR = 29.7%-69.2%) in public-referral hospitals, and 47.1% (IQR = 24.4%-71.5%) in private hospitals (**Table 2**). The differences decreased but still remained large when we restricted the analyses to hospitals with larger delivery volumes (Table S3 in the **Online Supplementary Document**). They decreased further to 43.0% (IQR = 19.0%-62.0%), 36.8% (IQR = 29.8%-66.6%), and 38.9% (IQR = 27.1%-66.0%), respectively, when analyses were restricted to the hospitals with over 1000 deliveries occurring annually, and to 40.9% (IQR = 17.9%-58.8%), 29.3% (IQR = 30.1%-59.4%) and 33.4% (IQR = 30.3%-63.7%), respectively, when restricted to the hospitals with over 3000 deliveries. Annual delivery volume was a crucial proxy for underlying health facility factors [25].

As expected, the largest variations within hospital types occurred in the western region of China, where the difference was 54.0% (IQR = 8.2%-62.2%) in public-non-referral, 42.9% (IQR = 25.2%-68.1%) in public-referral, and 51.7% (IQR = 19.7%-71.4%) in private hospitals. Furthermore, we observed the largest difference of 60.0% (IQR = 10.8%-70.8%) for public non-referral hospitals in Sichuan province, 47.5% (IQR = 31.6%-79.1%) for public-referral hospitals in Inner Mongolia and 76.7% (IQR = 11.0%-87.7%) for private hospitals in Xinjiang province, all of which were located in the western region (Table S4 in the **Online Supplementary Document**).

The geographic factors, including province and city of hospital location, appear to have accounted for most of the hospital variation in caesarean delivery rates (Table S5 in the **Online Supplementary Document**). For example, partial *R^2^* for hospital location was 0.606 in public-non-referral hospitals, indicating that 60.6% of hospital variation in caesarean delivery rates could be explained by the province and city of hospital location. For public-referral and private hospitals, the results were similar: partial *R^2^* for hospital location were 0.598 and 0.574, respectively.

## DISCUSSION

In this national data involving 38 517 196 deliveries from 7085 hospitals spreading throughout 31 provinces of mainland China, caesarean delivery rates in public-non-referral hospitals, public-referral hospitals, and private hospitals remained steady from 2016 through 2020, plateauing at over 40%. The relatively stable trend in rates over time was consistent with previously reported 2008-2018 national county-level aggregated data from the National Maternal and Child Health Statistics of China [3]. The overall average rate in public-non-referral hospitals, which were mainly handling uncomplicated pregnancies, was over two times the optimal rate of 15%-19% [26,27] and significantly higher than the optimal rate for Chinese population (28.5%) [28]. Even the rate in the underdeveloped western region of China (35.5%) was far beyond the threshold value. The high rates might be partly explained by repeated caesarean deliveries in multiparous women whose previous childbirths were done through caesarean deliveries following the enactment of the universal two-child policy in January 2016. For these women delivering the second child, caesarean delivery was more likely performed twice, owing to medical indications or safety concerns [19].

Caesarean delivery rates differed markedly across hospital types. We observed the highest rate in public-referral hospitals (47.0%) with a capacity of dealing with complicated pregnancies such as multiple pregnancies and pregnancies with severe morbidities that were more likely to end with a caesarean delivery [29,30]. We observed the second highest rate (45.8%) private hospitals – an overwhelming majority of which were non-referral, a figure much closer to that for public-referral rather than public-non-referral hospitals (40.3%). Higher caesarean delivery rates in private vs public hospitals were reported in other countries, including India, Brazil, and Australia [12,15,16]. In India and Brazil [12,15], the rates were only 11.9% and 31.0% for public hospitals, respectively, increasing to 40.9% and 72.1% for private hospitals. In Australia [16], the difference in the rates between public (26.9%) and private hospitals (48.0%) was also large, persisting over all ten subgroups of women categorized by Robson classification. The variation between private and public hospitals might be attributed to several factors, including governmental supervision, health insurance and health system, as well as patients’ clinical characteristics, preferences, or wealth [31-33]. In high-income countries like Australia [16], the variation could be partly explained by patients’ clinical characteristics like parity and foetal presentation or health system factors like professional training on vaginal births for women with caesarean scars [16,29]. In middle-income countries like Brazil [15], the large variation was largely explained by health system factors like financial incentives rather than patients’ clinical characteristics or preferences [15]. In low-income countries like India [12], the very large variation not due to the patients’ clinical characteristics, but more likely due to other aforementioned factors, particularly inadequate governmental supervision, facility capacities besides financial incentives, and patients’ wealth [12,33]. In China, private hospitals are inclined to serve patients who prefer caesarean delivery and are affordable, possibly to some extent deviating from health authorities’ improving in-hospital delivery [8,33]. Additionally, caesarean delivery operation per se costs approximately two times more than a vaginal delivery [23]. The overuse of caesarean delivery is seemingly also present in mainland China, giving cause for concerns, especially due to the growing number of private hospitals.

The overall variation across hospital types was not present in the northeastern region of China, where it disappeared unexpectedly, with extremely high rates approaching 60% in all three types of hospitals. The caesarean delivery rate in public-non-referral hospitals was much higher than that in public-referral hospitals located in 14 super cities of the other regions of China. Moreover, the rates in Heilongjiang province of the northeastern region of China were over 62.5%, with the highest rates in public-non-referral rather than in public-referral or private hospitals. Despite this, maternal mortality rates in the region remained the highest for referral hospitals and the second highest for non-referral hospitals, close to or right behind those in the underdeveloped western region of China, indicating extremely high caesarean rates were not linked with decreased maternal mortality. A small-scale study including 16 hospitals of only Jiangsu Province of China also showed a higher rate in non-referral hospitals (44.7%) vs referral hospitals (40.4%) [34], yet such findings were not reported internationally. Our findings appeared unique for China. The extremely high caesarean delivery rates in the northeastern region of China, where the difference between hospital types was almost non-existent, are a cause for concern. Comprehensive measures are needed to mitigate the situation for maternal and child health, since our findings could not be accounted for by any single factor [17,35].

Caesarean rates within each of hospital types also differed markedly. Currently, no studies reported on existing variations in China and other developing countries. For developed countries, the largest difference in the rates between hospitals was 62.8% in the United States [36],18.3% in the United Kingdom [29], and 35.6% in Australia [37]. In our study, the 5th and 95th percentiles of caesarean delivery rates differed significantly at 52.0% in public-non-referral hospitals, 39.5% in public-referral hospitals, and 47.1% in private hospitals. The variation within all three hospital types, despite the observed difference between the 5th and 95th percentiles but not the range of the caesarean rates, was larger than that in the United Kingdom and Australia. Overall, the largest 95 th percentile of rates in our study was in private hospitals in rural area of the northeastern region of China, and the lowest 5th percentile was in public-non-referral hospitals in the rural areas of the western region, resulting in an overall difference of 82.9% that was also much larger than what was reported in the United States. In our study, the variation persisted in all subgroups, and was more pronounced in the western region of China; yet even within this region, the variation differed largely, where the differences in rates between the 5th and 95th percentiles of caesarean delivery rates for three hospital types ranged from 51.5% to 64.6% in the rural areas vs only 18.9% to 24.0% in the super cities, indicating a co-existence of overuse and underuse of caesarean deliveries in the region. The large within-hospital-type variation might reflect disparities in social-economic development, health infrastructure, professional capabilities, or other relevant factors that are unevenly distributed geographically. Similar to previous studies in China [17] and the United States [38], geographic factors played a role. In our study, hospital location, i.e., a province and city within the province, accounted for over 57% of the variation.

### Study strengths and limitations

Across- and within-hospital-type variations in caesarean delivery rates for public-non-referral, public-referral, and private hospitals throughout mainland China have not been documented. The NCIS collected large-scale national hospital-level data covering all 31 provinces of mainland China. The data collection system was established in 2011 by all levels of governmental health authorities, participating hospitals were selected in accordance with an elaborate sampling strategy to be representative of national and local levels, and information was collected from clinical records, with various measures to assure data quality at all levels. Hospitals with less than 100 deliveries per year were excluded to robustly estimate hospital-specific rates. Unlike prior studies, the difference between the 5^th^ and 95^th^ percentiles of caesarean delivery rates rather than a range in our study was used to characterize the variation, making results robust and less likely to have been affected by extreme values or outliers.

The study had several limitations. The NCIS data was aggregated hospital-level rather than patient-level data. Although we could not adjust for individual clinical characteristics in estimating caesarean delivery rates, we examined the reported variation by hospital types that could largely reflect cross-hospital-type clinical heterogeneity in patients’ populations. For example, advanced maternal age, multiple pregnancies, and severe morbidities, together with maternal deaths, were more likely to have been observed in public-referral hospitals rather than in other hospitals. The NCIS data were hospital- rather than population-based, and so the overall rate could not necessarily reflect caesarean delivery occurrence in populations, largely due to the restrictions exerted by the hospital selected. The NCIS caesarean delivery rates for selected hospitals have not yet been validated via on-site visits, but did not differ materially from those estimated by prior hospital-based studies, after taking sampling strategies into account. We classified the hospital types according to the registered types updated last during 2016-2020. Despite it being unlikely that the hospital types changed over a short period, a possibility that a non-referral hospital was promoted to a referral could not be completely excluded, possibly leading to an underestimation of rates for referral hospitals. Caution is needed when applying our results from aggregated data to individual level, as the associations between geographical factors and hospital variations in caesarean delivery rate might be biased by other confounders, such as social-economic factors, physicians’ capabilities, and patients’ clinical characteristics [29,31-33]. Although we attempted to explore potential sources, this was not the primary aim of our study.

## CONCLUSIONS

Between 2016 and 2020, the annual caesarean delivery rates in three types of hospitals in mainland China plateaued at over 40%. Variation across hospital types was pronounced, with the highest rate either in public-referral or private hospitals, except for the northeastern region where variation was nearly non-existent, despite the extremely high rates. Variation between hospital types was also pronounced, especially for public-non-referral or private hospitals located in rural areas of the western region of mainland China.

## Additional material


Online Supplementary Document
