# Supplementary material for: Hospital variations in caesarean delivery rates: An analysis of national data in China, 2016-2020
Source: J Glob Health. 2023 Apr 7;13:04029. doi: 10.7189/jogh.13.04029 (PMC10078857; doi:10.7189/jogh.13.04029)
Supplement: Online Supplementary Document [file jogh-13-04029-s001.pdf]

Online Supplementary Document

Title: Hospital variations in cesarean delivery rates: an analysis of national data in China, 2016–2020

Authors: Shaohua Yin, Yubo Zhou, Pengbo Yuan, Yuan Wei, Lian Chen, Xiaoyue Guo, Hongtian Li, Jie Lu, Lin Ge, Huifeng Shi, Xiaoxia Wang, Luyao Li, Jie Qiao, Dunjin Chen, Jianmeng Liu, Yangyu Zhao

Appendix

Contents

Supplementary Tables 2
Supplementary Figures 7

Supplementary Tables

Table S1. Hospital sampling strategy in the NCIS.

**Level 1**	**Level 2**	**Level 3**	**Inclusion criteria**
Public hospitals	Central government hospitals		All
	Provincial or municipal hospitals	Hospitals affiliated with university	All
		Hospitals without university affiliation	1–5 hospitals per province
	City hospitals		1–2 hospitals per city, and 1–2 hospitals per district for municipality
	County hospitals	Non-referral hospitals	3–4 hospitals per city
		Referral hospitals	A percentage of hospitals based on the ratio of number of city-level to county-level hospitals
Private hospitals			All

Table S2. Cesarean delivery rates by hospital types, 2016–2020.

	2016	2017	2018	2019	2020	Total
**Total deliveries, No.**	6 855 889	6 859 428	9 383 778	8 739 797	6 678 304	38 517 196
**Predicted mean rate, % (No.)***	42.9 (2 941 062)	43.1 (2 957 978)	44.1 (4 135 482)	44.5 (3 889 211)	44.7 (2 986 498)	43.9 (16 910 231)
**Mean rate, % (No.)**	42.9 (2 941 062)[a]	43.2 (2 962 721)	43.4 (4 069 983)	43.9 (3 840 154)	43.9 (2 930 485)	43.5 (16 744 405)
**Median rate (IQR), %**	43.2 (34.6–53.1)	44.0 (35.5–54.2)	43.9 (34.7–53.9)	44.6 (35.4–54.4)	44.3 (35.2–54.0)	44.0 (35.1–53.9)
Public-nonreferral hospitals	39.8 (30.6–49.7)	41.2 (31.8–51.3)	40.8 (31.1–51.2)	41.7 (31.8–51.9)	41.2 (32.0–51.2)	41.0 (31.5–51.1)
Public-referral hospitals	46.5 (39.3–55.7)	47.2 (40.2–56.3)	47.7 (39.8–56.9)	47.2 (39.9–56.5)	48.0 (40.5–57.1)	47.4 (39.9–56.5)
Private hospitals	45.5 (37.2–55.4)	45.5 (36.8–55.6)	44.8 (36.0–54.9)	47.2 (37.6–55.8)	46.6 (37.3–56.8)	45.8 (36.9–55.7)

IQR=interquartile range.

*The rates were calculated from the imputed data estimated using liner regression models established for each hospital.

Table S3. Median and difference (P95 minus P5)* of cesarean delivery rates across hospital types by geographic region and hospital delivery volume, 2016–2020.

		Public-nonreferral hospitals		Public-referral hospitals		Private hospitals	
**Geographic region**	**Hospital delivery volume**	Median (IQR)	Difference (P5–P95)	Median (IQR)	Difference (P5–P95)	Median (IQR)	Difference (P5–P95)
Northeastern†	<1000	59.2 (53.1–71.8)	41.3 (42.2–83.6)	62.2 (55.1–69.1)	33.9 (46.5–80.4)	61.0 (53.4–69.5)	41.0 (38.9–79.9)
	1000–2999	56.9 (48.4–64.4)	34.4 (43.9–78.3)	57.4 (51.2–64.9)	35.1 (40.3–75.4)	56.2 (48.7–63.6)	25.6 (45.2–70.8)
	3000–4999	..†	..†	58.7 (54.7–65.6)	37.5 (38.3–75.8)	..†	..†
	≥5000	..†	..†	47.2 (39.8–57.2)	45.2 (34.2–79.4)	..†	..†
Eastern	<1000	43.1 (32.5–53.5)	50.6 (20.1–70.7)	44.3 (37.3–54.0)	47.2 (29.2–76.4)	46.2 (36.8–56.2)	48.5 (24.5–73.0)
	1000–2999	39.1 (32.4–48.1)	39.2 (24.3–63.5)	44.9 (38.7–52.0)	30.5 (30.5–61.0)	44.0 (36.9–53.7)	42.9 (27.1–70.0)
	3000–4999	38.2 (31.1–46.2)	35.9 (22.8–58.7)	42.3 (38.5–51.9)	30.0 (30.9–60.9)	41.3 (37.7–51.3)	42.1 (27.5–69.6)
	≥5000	35.3 (29.3–43.9)	41.0 (16.6–57.6)	41.7 (37.0–45.9)	26.7 (30.1–56.8)	39.8 (33.0–44.4)	23.5 (28.9–52.4)
Central	<1000	40.6 (31.4–50.8)	50.1 (20.1–70.2)	50.4 (42.2–60.5)	37.2 (33.9–71.1)	44.4 (33.3–54.8)	42.9 (25.0–67.9)
	1000–2999	43.1 (35.5–51.7)	39.0 (24.5–63.5)	49.9 (41.3–57.5)	35.2 (35.0–70.2)	45.2 (37.6–52.5)	32.7 (30.1–62.8)
	3000–4999	43.3 (35.4–49.9)	33.3 (27.3–60.6)	48.2 (41.8–57.9)	31.4 (35.3–66.7)	47.4 (40.6–54.3)	29.9 (37.0–66.9)
	≥5000	40.6 (36.0–45.1)	34.2 (24.8–59.0)	45.9 (39.5–52.5)	31.2 (31.6–62.7)	42.3 (32.4–44.5)	35.0 (17.0–52.1)
Western	<1000	37.5 (23.5–49.9)	62.1 (3.1–65.1)	49.2 (38.5–57.9)	53.2 (24.9–78.1)	45.5 (33.4–56.0)	53.8 (19.2–73.1)
	1000–2999	34.1 (26.6–43.7)	44.1 (14.5–58.6)	48.3 (40.3–56.3)	42.8 (24.7–67.5)	43.3 (36.1–51.2)	36.4 (23.5–59.9)
	3000–4999	31.6 (24.2–43.3)	43.8 (12.5–56.3)	46.5 (39.9–54.8)	41.9 (26.1–68.0)	38.4 (35.5–46.3)	29.2 (24.2–53.4)
	≥5000	28.9 (23.4–39.7)	35.2 (16.3–51.5)	44.0 (38.8–51.0)	31.4 (28.4–59.7)	..†	..†

IQR=interquartile range. P5=5th percentile. P95=95th percentile. Data are percentiles of cesarean delivery rates (%).

*Difference was calculated by subtracting the 5th percentile from the 95th percentile.

†The values could not be estimated due to limited number of hospitals.

Table S4. Difference (P95 minus P5)* of cesarean delivery rates across hospital types by province†

	Public-nonreferral hospitals			Public-referral hospitals			Private hospitals		
	P5	P95	Difference	P5	P95	Difference	P5	P95	Difference
Anhui	25.0	47.9	22.9	34.3	58.4	24.1	25.3	54.7	29.4
Beijing	30.7	55.3	24.6	29.9	52.6	22.7	28.8	50.3	21.5
Chongqing	35.4	58.0	22.6	41.5	63.0	21.5	43.5	67.5	24.0
Fujian	21.6	43.7	22.1	28.3	50.5	22.2	20.7	52.9	32.2
Gansu	9.2	49.4	40.2	20.2	53.4	33.2	24.1	50.0	25.9
Guangdong	17.1	45.4	28.3	24.7	48.2	23.5	17.9	50.3	32.4
Guangxi	15.1	41.6	26.5	24.7	49.8	25.1	18.9	39.3	20.4
Guizhou	16.0	56.1	40.1	26.1	68.1	42.0	18.0	71.4	53.4
Hainan	0	45.1	45.1	26.9	60.9	34.0	22.8	52.4	29.6
Hebei	25.3	75.5	50.2	36.9	73.5	36.6	25.5	80.3	54.8
Heilongjiang	43.4	83.8	40.4	45.6	79.9	34.3	48.0	84.0	36.0
Henan	28.7	67.7	39.0	43.9	73.1	29.2	35.5	66.9	31.4
Hubei	37.8	76.3	38.5	47.4	72.9	25.5	47.9	83.0	35.1
Hunan	24.5	57.1	32.6	36.1	61.4	25.3	27.8	64.2	36.4
Inner Mongolia	24.2	74.3	50.1	31.6	79.1	47.5	37.1	75.7	38.6
Jiangsu	32.1	68.6	36.5	36.8	66.3	29.5	32.6	70.0	37.4
Jiangxi	18.5	54.1	35.6	29.9	55.7	25.8	14.6	61.1	46.5
Jilin	39.6	61.4	21.8	38.5	75.8	37.3	29.1	74.4	45.3
Liaoning	43.8	84.1	40.3	40.3	80.1	39.8	34.7	76.3	41.6
Ningxia	0	42.8	42.8	30.7	56.1	25.4	28.1	52.1	24.0
Qinghai	0	28.7	28.7	6.2	47.3	41.1	28.5	51.2	22.7
Shaanxi	23.3	52.8	29.5	38.5	59.9	21.4	29.0	58.0	29.0
Shandong	28.6	67.5	38.9	36.6	63.7	27.1	34.6	71.5	36.9
Shanghai	31.0	64.8	33.8	38.0	66.1	28.1	28.0	63.9	35.9
Shanxi	15.9	53.2	37.3	29.3	60.1	30.8	26.4	66.5	40.1
Sichuan	10.8	70.8	60.0	42.2	73.2	31.0	33.0	82.7	49.7
Tianjin	35.5	60.1	24.6	42.3	63.0	20.7	24.8	73.0	48.2
Tibet	0	19.7	19.7	6.6	33.6	27.0	..‡	..‡	..‡
Xinjiang	16.8	60.1	43.3	34.9	68.0	33.1	11.0	87.7	76.7
Yunnan	8.6	42.5	33.9	24.9	58.3	33.4	15.8	53.4	37.6
Zhejiang	26.4	52.8	26.4	32.9	55.5	22.6	33.0	54.5	21.5

P5=5th percentile. P95=95th percentile. Data are percentiles of cesarean delivery rates (%).

*Difference was calculated by subtracting the 5th percentile from the 95th percentile.

†Northeastern region included Heilongjiang, Liaoning, and Jilin. Eastern region included Beijing, Tianjin, Hebei, Shandong, Jiangsu, Shanghai, Zhejiang, Fujian, Guangdong, and Hainan. Central region included Shanxi, Henan, Anhui, Jiangxi, Hubei, and Hunan. Western region included Chongqing, Sichuan, Guizhou, Yunnan, Tibet, Guangxi, Shaanxi, Gansu, Qinghai, Ningxia, Xinjiang, and Inner Mongolia.

‡The values could not be estimated due to limited number of hospitals.

Table S5. Partial R^2 of maternal, hospital, and geographic factors on variation in cesarean delivery rates, 2016–2020*.

	**Public-nonreferral hospitals (n=4103)**		**Public-referral hospitals (n=1805)**		**Private hospitals (n=1177)**	
	Partial R^2	Rank	Partial R^2	Rank	Partial R^2	Rank
**Maternal factors**	**0.058**		**0.074**		**0.027**	
Hospital-specific percentage of AMA women	0.004	6	0.009	7	0.003	8
Hospital-specific percentage of multiparous women	0.017	4	0.018	6	0.009	5
Hospital-specific percentage of women with multiple pregnancies	0.004	7	0.021	3	0.004	7
Maternal mortality	..†		0.004	10	..†	
Maternal severe morbidity rate	..†		0.003	11	..†	
Hospital-specific percentage of macrosomia	0.033	3	0.019	5	0.011	4
**Hospital factors**	**0.008**		**0.029**		**0.007**	
Hospital-specific percentage of women with epidural anesthesia	0.002	9	0.001	12	0.002	9
Density of midwives	0.002	10	0.006	9	0.001	10
Hospital-specific annual delivery volume	0.004	8	0.022	4	0.004	6
**Geographic factors**	**0.617**		**0.607**		**0.590**	
Province of hospital location	0.218	2	0.225	2	0.181	2
City of hospital location	0.388	1	0.373	1	0.393	1
Urbanization level	0.011	5	0.009	8	0.016	3
**Combined**	**0.683**		**0.710**		**0.624**	

AMA= advanced maternal age.

*Partial R^2 were derived from multivariate linear regression models adjusted for maternal factors (hospital-specific percentage of AMA women, multiparous women, women with multiple pregnancies, and macrosomia, maternal mortality, and maternal severe morbidity rate), hospital factors (hospital-specific percentage of women with epidural anesthesia, density of midwives, and hospital-specific annual delivery volume), and geographic factors (province and city of hospital location, and urbanization level).

†Factor did not show a significant relationship with cesarean delivery rate in univariate linear regression.

Supplementary Figures

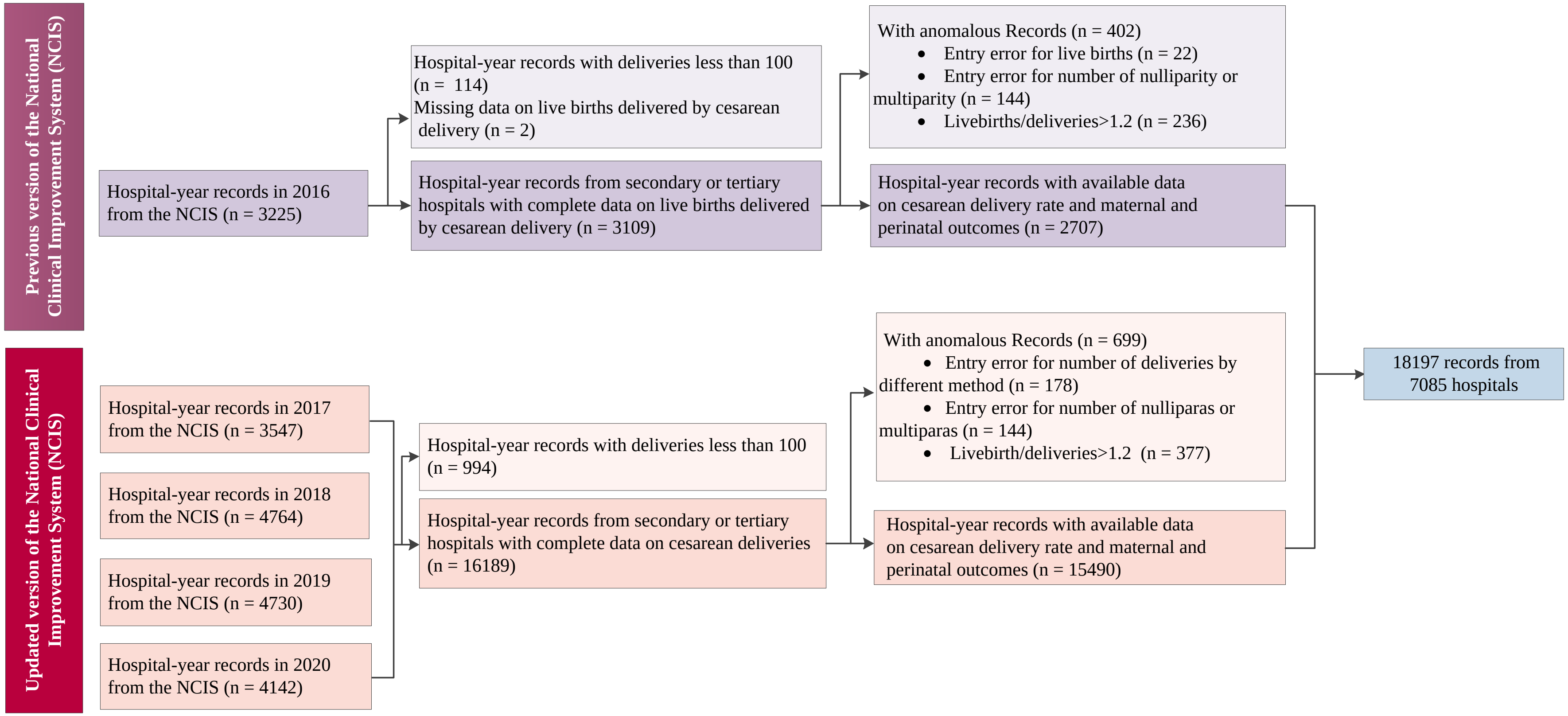

Figure S1. Flowchart of hospital-year records in the NCIS, 2016–2020.

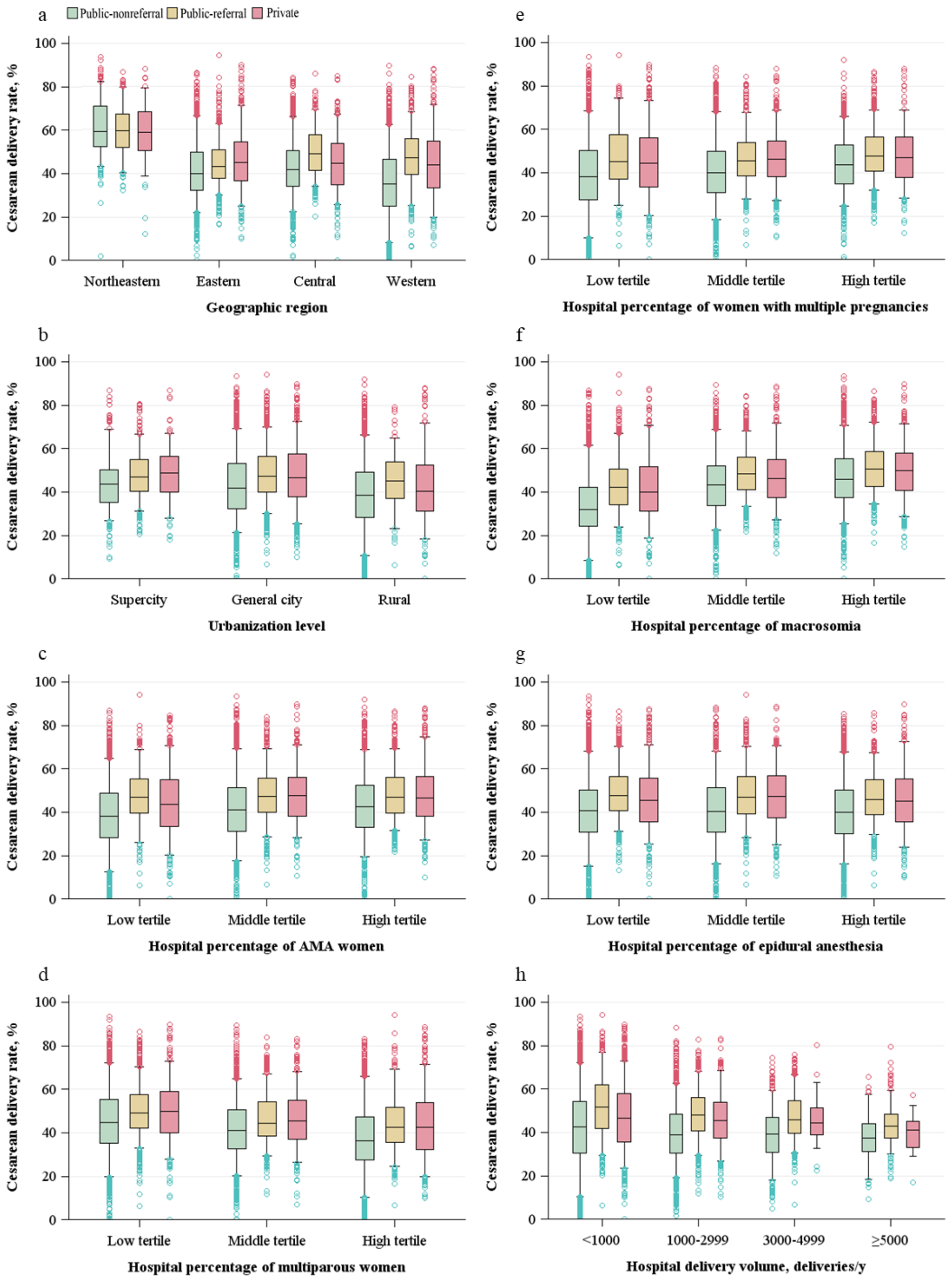

Figure S2. Cesarean delivery rates across hospital types by geographic region (a), urbanization level (b), hospital percentage of AMA women (c), hospital percentage of multiparous women (d), hospital percentage of women with multiple pregnancies (e), hospital percentage of macrosomia (f), hospital percentage of epidural anesthesia (g), and hospital delivery volume (h), 2016–2020. For each box-and-whisker plot, the horizontal bar indicates the median, the upper and lower limits of the boxes the interquartile range, and the ends of the whiskers from bottom of box to top indicate the 5th percentile and the 95th percentile. The green and red circles respectively represent the rates less than 5th percentile or greater than 95th percentile.

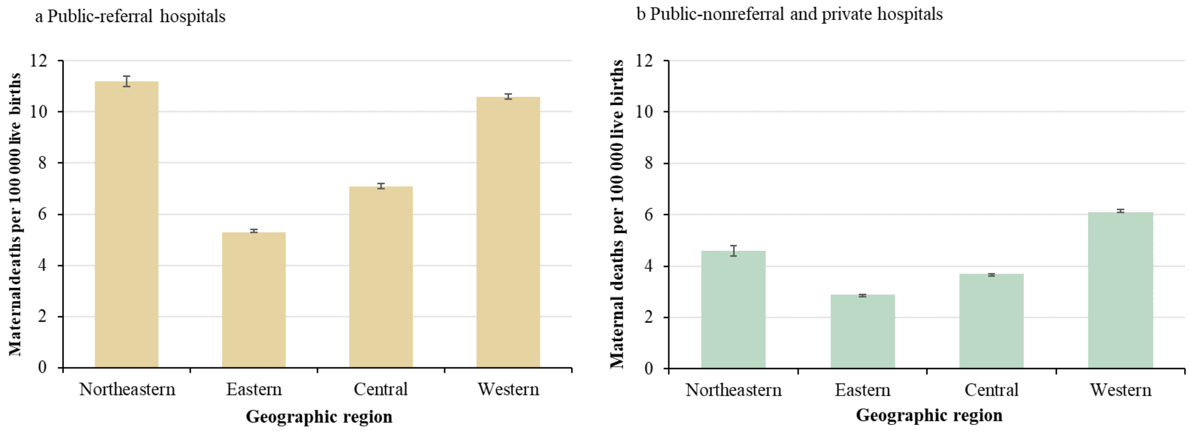

Figure S3. Maternal mortality (95% CI) across geographic region in public-referral hospitals (a), and public-nonreferral and private hospitals (b), 2016–2020. Each bar indicates the maternal mortality. The error bars indicate the 95% confidence interval (95%CI).
